# Joubert Syndrome: A Rare Case of Two Sudanese Sisters With Neurodevelopmental Delays and Diagnostic Challenges

**DOI:** 10.1002/ccr3.70733

**Published:** 2025-07-30

**Authors:** Ahmed Alshafei Elmahi Ahmed, Mehad Mortada BadrAlden Ahmed, Aisha Gameraldeen Abdalrhim Ibrahim, Arafa Mubarak Abotalib Aref, Thowiba Mohammed Abdalla Saidahmed, Osman Elshazali Osman Abd Elaziz, Zahra Abdalla Ahmed Neel, Mohammed Musa Abozaid Alkarar, Tarig Mohamed Nourallah Altegani, Amjed Abdu Ali Mohammed, Mohammed Hammad Jaber Amin

**Affiliations:** ^1^ Faculty of Medicine The National Ribat University Khartoum Sudan; ^2^ Faculty of Medicine and Health Science Nile Valley University Atbara Sudan; ^3^ Faculty of Medicine Sheikh Abdullah Al‐Badri University Berber Sudan; ^4^ Faculty of Medicine and Health Science Omdurman Islamic University Khartoum Sudan; ^5^ Faculty of Medicine Khartoum University Khartoum Sudan; ^6^ Sudanese Medical Specialty Board Khartoum Sudan; ^7^ Faculty of Medicine Alzaiem Alazhari University Khartoum Sudan

**Keywords:** Africa, developmental milestones, genetic disorder, Joubert syndrome, magnetic resonance imaging, molar tooth sign, Sudan

## Abstract

Joubert Syndrome's rarity and diagnostic complexity, especially in Sudan, pose significant challenges in low‐resource settings. Sibling cases with neurodevelopmental delays and MRI‐confirmed molar tooth sign highlight the urgent need for heightened clinical suspicion, accessible neuroimaging, and genetic counseling to address underdiagnosis in underrepresented populations.

**Abbreviations**
AS: autosomal recessive
CMV: cytomegalovirus
CNS: central nervous system
EPI: expanded programme on immunization
GCS: Glasgow coma scale
JS: Joubert syndrome
JSRD: Joubert syndrome‐related disorders
MRI: magnetic resonance imaging
PNS: peripheral nervous system
SAM: sever acute malnutrition

## 1 Introduction

Joubert syndrome (JS) is an autosomal recessive rare genetic disease that occurs in approximately 1 in 80,000 to 1 in 100,000 individuals [1] and is primarily inherited in an autosomal recessive fashion. Classic Joubert syndrome (JS) presents with distinctive clinical findings, including hypotonia, ataxia, oculomotor apraxia, and intellectual disability [2]. Besides these core neurological features, JS also shows an enormous range of phenotypic presentations, ranging from retinal, renal, hepatic, oral‐facial‐digital symptoms and signs, to classical neurological symptoms, according to the subtype of JS. Multiple forms of JS have already been reported, and new ones are still being added in recent years [3, 4].

JS belongs to the category of illnesses known as ciliopathies. The primary cilium, a crucial subcellular structure, plays a significant role in the development of neural circuits. Mutations in genes responsible for the primary cilium result in a range of symptoms linked to central nervous system (CNS) dysfunction [5]. While the primary cilium is essential for CNS function, it is also critical for the development of the peripheral nervous system (PNS) in living organisms [6]. Despite this, abnormalities in the PNS have not been documented in JS patients to date.

Traditionally, diagnosis of JS is made on typical clinical presentation and some radiological features, such as the “molar tooth sign.” However, recent observations have shown that some JS patients may present with normal or near‐normal brain imaging results [7]. Genetic analysis is increasingly being employed for diagnosing JS and is promising as a diagnostic test per se, particularly in rare cases [8].

Epidemiologic information, such as population‐based prevalence rates, is lacking in JS case reports. Due to the fact that the condition usually manifests as delayed milestones of development, most cases are likely undiagnosed during childhood. In association with developmental delays and respiratory distress, suspicion of JS is evoked [9]. Maria et al. found the mean age at diagnosis was 33 months [10]. Though most children with this syndrome survive infancy and adulthood [11], early diagnosis is needed to initiate early multidisciplinary management, including medical treatment and rehabilitation.

Arab populations exhibit significant ethnic diversity, with consanguinity rates ranging from 20% to 60%, leading to a high prevalence of autosomal recessive (AR) disorders. Additionally, isolated communities with extreme levels of inbreeding and founder mutations are frequently observed [12, 13, 14]. A study reviewed 70 families with JS and JSRDs from Arabic countries that have been analyzed at the molecular level, compiling the identified mutations. The findings demonstrate that Joubert syndrome (JS) and related Joubert syndrome‐related disorders (JSRDs) exhibit significant genetic heterogeneity within Arab populations, with 53 mutations identified across 15 genes, including 13 probable founder mutations unique to the region [15].

JS has been documented globally; its prevalence and genetic profile in Africa and Arab countries, particularly Sudan, remain poorly understood due to limited reporting and research. This case report presents two Sudanese sisters with JS, highlighting the clinical and genetic aspects of the syndrome while emphasizing its scarcity in Africa and Arab populations, including Sudan.

## 2 Case Presentation

### 2.1 Case One

A 2‐year‐old female child of consanguineous parents was presented at the clinic with global developmental delay since childhood, as reported by her mother, and abnormal eye movements since the age of one. The developmental delay was manifested by not sitting without support, walking, or speaking, but a social smile on hearing sounds since 2 months of life. Her natal, pregnancy, and postnatal history were unremarkable.

Her medical history in the past included two previous admissions to the hospital: first at 40 days of age for fits and fever, diagnosed as meningitis, and the second admission due to severe acute malnutrition with gastroenteritis clinically diagnosed as postmeningitic cerebral sequelae. She was fully vaccinated under the Expanded Program on Immunization (EPI) and had a positive family history of the same illness in her younger sister.

**FIGURE 1:**
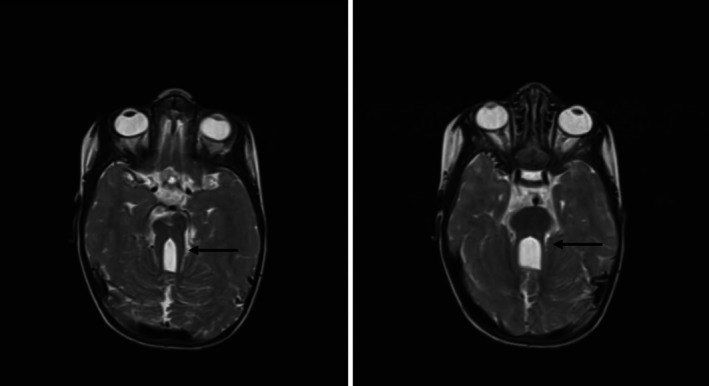
Brain MRI findings were consistent with Joubert syndrome, demonstrating the characteristic ‘molar tooth sig’, which arises from cerebellar vermis hypoplasia, elongated superior cerebellar peduncles, and a deep interpeduncular cistern. This radiological hallmark supported the clinical diagnosis of Joubert syndrome in our patient.

On presentation, the patient was ill but not dysmorphic with bossing of the front. Her anthropometric and vital signs measurements were not appropriate for her age: weight 6.5 kg (< 3rd percentile), height 77 cm (< 3rd percentile), head circumference 47 cm (3rd–50th percentile), and MUAC 12 cm. Neurologic exam was positive for Glasgow Coma Scale (GCS) of 15/15, nystagmus, reactive pupils to light, hypotonia of all extremities, normal reflexes in all extremities, no fasciculations, power grade 5, and cranial nerve and back exam normal. The remainder of the systemic and workups were unremarkable. Hematological findings on examination were raised total white blood cells (lymphocyte‐predominant), hypochromic microcytic anemia, and normal platelets. Bone profile and renal were normal, and abdominal ultrasound reports were negligible. Brain MRI was showing thickened elongated superior cerebellar peduncle with midline cleft creating molar tooth appearance along with inferior vermian hypoplasia—features observed in Joubert syndrome (as follows in the image) (Figure 1). Genetic testing was not carried out due to ongoing socioeconomic challenges in Sudan, including conflict‐related disruptions, financial constraints, and lack of local facilities offering molecular diagnostics for ciliopathies.

The patient was managed by a multidisciplinary team of neuropaediatricians, physiotherapists, and general pediatricians. Regular follow‐up was provided to the patient at a nutritional rehabilitation center.

*Clinical features*: (Table 1).

**TABLE 1:** Clinical features table.

Feature	Case 1	Case 2	JS hallmark
Global developmental delay	Yes	Yes	Common
Nystagmus	Yes	Yes	Common
Hypotonia	Yes	Yes	Common
Molar tooth sign (MRI)	Yes	Yes	Pathognomonic
Hepatosplenomegaly	No	Yes	Seen in TMEM67 subtypes
Microcytic anemia	Yes	No	Non‐specific

### 2.2 Case Two

A 9‐month‐old female sibling of the aforementioned case presented as a co‐patient, exhibiting clinical features similar to her sister, including nystagmus, frontal bossing, and developmental delay. Her pregnancy, natal, and postnatal periods were uneventful. Her mother reported global developmental delay, manifested as an inability to sit without support and only a social smile. She had a history of recurrent hospital admissions and blood transfusions, though no definitive diagnosis was reached. She was fully vaccinated per Sudan's Expanded Programme on Immunization (EPI).

On examination, the patient appeared unwell but not dysmorphic, with frontal bossing. Vital signs and anthropometric measurements were as follows: weight 3.95 kg (< 3rd percentile), length 57 cm (< 3rd percentile), head circumference 43 cm (3rd–5th percentile), and MUAC 12 cm. Neurological assessment revealed a Glasgow Coma Scale (GCS) of 15/15, nystagmus, pupils reactive to light, normal muscle tone and reflexes in all limbs, power grade 5, and unremarkable cranial nerve and back examinations. Abdominal examination showed distention with full flanks, a palpable liver 4 cm below the costal margin (nontender), and a spleen 6 cm below the costal margin, with no lymphadenopathy. Other systemic examinations were unremarkable.

**FIGURE 2:**
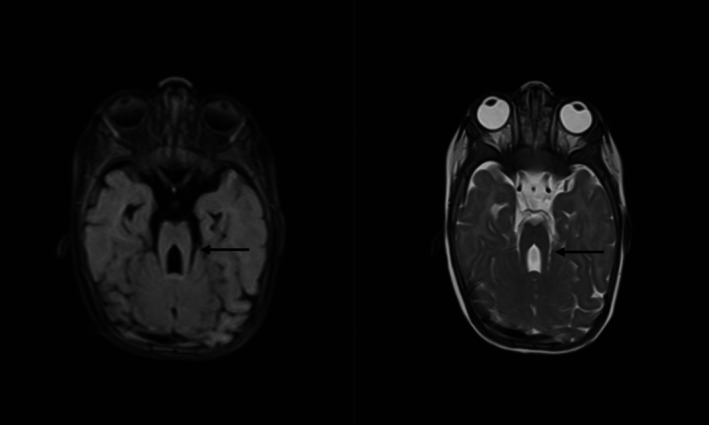
Brain MRI findings were consistent with Joubert syndrome, demonstrating the characteristic ‘molar tooth sig’, which arises from cerebellar vermis hypoplasia, elongated superior cerebellar peduncles, and a deep interpeduncular cistern. This radiological hallmark supported the clinical diagnosis of Joubert syndrome in our patient.

Laboratory investigations demonstrated elevated total white blood cells (lymphocyte‐predominant), microcytic hypochromic anemia, thrombocytopenia, and a high reticulocyte count. Hemoglobin electrophoresis and renal profile were normal, while liver function tests showed elevated enzymes. TORCH serology was positive for IgG antibodies to cytomegalovirus (CMV) and rubella. Brain MRI revealed a thickened and elongated superior cerebellar peduncle with a midline cleft, producing a molar tooth appearance, alongside inferior vermian hypoplasia consistent with Joubert syndrome (Figure 2). Genetic analysis could not be performed owing to regional instability from conflict, limited funding for advanced diagnostics, and absence of genetic testing infrastructure in Sudan.

The patient was managed by a multidisciplinary team, including physiotherapists, neuropaediatricians, and general pediatricians, and received ongoing follow‐up care at a nutritional stabilization center.

*Clinical features*: (Table 1).

### 2.3 Differential Diagnosis for the Two Cases of Suspected Joubert Syndrome

The molar tooth sign, consanguinity, and clinical features strongly support Joubert syndrome. Case 2 hepatosplenomegaly may reflect an extended ciliopathy phenotype (TMEM67 mutation) or comorbid malnutrition/infection. TORCH infections and metabolic disorders remain secondary considerations. Genetic testing (ciliopathy panel) is critical for definitive diagnosis (Table 2).

**TABLE 2:** Differential diagnosis for the two cases of suspected joubert syndrome.

Diagnosis	Nature (clinical/radiological/laboratory)	Key features	Relation to JS
Joubert syndrome	Clinical + Radiological	Molar tooth sign, hypotonia, nystagmus	Confirmed by MRI in both cases
TORCH infections	Laboratory + Clinical	CMV/rubella IgG positivity, thrombocytopenia	Ruled out by absence of IgM and atypical MRI
Pontocerebellar hypoplasia	Radiological	Cerebellar/pontine atrophy	Lacks molar tooth sign
Dandy–Walker Malformation	Radiological	Cystic 4th ventricle, vermian hypoplasia	No midline cleft or elongated peduncles
Mitochondrial disorders	Clinical + Laboratory	Liver dysfunction, developmental delay	No lactate elevation or basal ganglia lesions

## 3 Discussion

Joubert syndrome (JS) is an autosomal recessive neurodevelopmental disorder that is characterized by partial or complete hypoplasia of the cerebellar vermis, the pathognomonic molar tooth sign on neuroimaging, and a variable clinical pattern that encompasses hyperpnea, hypotonia, neurological impairment, oculomotor apraxia, ataxia, and delayed achievement of developmental milestones. The syndrome was first described in 1968 with the typical neurological malformations and episodic tachypnea [16]. JS remains a seldom‐reported illness, and the number of cases reported in the literature cumulatively totals approximately 200 through 2009 [17].

Joubert syndrome (JS) has been divided into eight subtypes: the classic and variants with retinal disease, renal disease, oculorenal disease, liver disease, oral‐facial‐digital anomalies, acrocallosal features, and asphyxiating thoracic dystrophy features [18]. While JS patients do not typically exhibit a characteristic or uniform facial phenotype, as seen in our patient, certain individuals may present with recognizable facial features, such as a protruding mandible, that may serve as an adjunctive diagnostic hint [19, 20]. In addition to clinical diagnosis, research directed at genotype–phenotype correlations offers valuable information about recurrence risks and allows for the prediction of disease expression in future generations [21, 22, 23].

To date, over 35 causative genes have been reported for Joubert syndrome, with only one of these genes located on the X chromosome [24, 25]. The disorder, which follows an autosomal recessive inheritance pattern, is largely accounted for by mutations in genes such as NPHP1, CEP290, and AHI1 [3]. These mutations of genes disrupt various signaling pathways, leading to abnormal neuronal cell proliferation and migration, and consequently, the diverse neurological and respiratory manifestations in affected individuals [16].

Joubert syndrome (JS) is more specifically defined by the presence of delayed developmental milestones, the molar tooth sign, and a batwing fourth ventricle configuration, along with brainstem and cerebellar vermis malformations. On the other hand, Joubert syndrome‐related disorders (JSRD) comprise patients who have the clinical and imaging features of JS, along with additional systemic features involving various organs. While the central nervous system (CNS) is the predominant site of involvement, extra‐CNS involvement most frequently involves the eyes, skin, esophagus, kidneys, liver, spleen, and other components of the gastrointestinal tract [26, 27, 28]. Given the multi‐organ involvement of the disorder, referral to interested subspecialties at the first presentation itself is essential for diagnosis at an early stage and for improved management, thus enhancing the quality of life. All organ systems must be systematically evaluated to accurately delineate the whole spectrum of the disorder. Diagnosis of Joubert syndrome (JS) typically entails the use of MRI scan, retinal examination, renal ultrasound, electroretinograms, and karyotyping, although most cases are diagnosed primarily on the grounds of radiographic findings [27].

There is no curative treatment for JS yet, and management remains supportive with symptomatic treatment using a multidisciplinary approach with cognitive, behavioral, occupational, and psychiatric interventions to manage the broad spectrum of manifestations of the disorder [17]. Patients are advised to undergo special language therapy, individualized educational programs to acquire appropriate skills, and annual screenings to monitor the progression of the disease [9]. In the given case, the patient experienced hypotonia and lack of coordination in voluntary movements. Following complete examination of all the organ systems and illustration of the characteristic molar tooth sign on MRI, the patient was diagnosed with pure JS. Such cases are extremely rare worldwide, extremely challenging to diagnose, and most frequently associated with severe disabilities due to the probability of late diagnosis. Furthermore, there are barriers to numerous patients being referred to subspecialists, such as neurologists, psychiatrists, and pediatricians, who are key to early diagnosis and treatment.

## 4 Conclusion

To the best of our knowledge, this is the first documented case of Joubert syndrome (JS) in Sudan and the second case reported from Africa. This was achieved with the demonstrated presence of the molar tooth sign, hypotonia, and delay in development. JS is a rare condition in Sudan, and the lack of cases from Africa reinforces the need for further awareness and access to diagnosis in low‐resource settings. Our assessment and management, facilitated by a multidisciplinary team, demonstrated the benefits of team care in managing a rare genetic condition. This publication developed the limited literature regarding JS globally and advocates for better healthcare systems to manage the underdiagnosed genetic conditions in Africa and Sudan specifically.

## Author Contributions

**Ahmed Alshafei Elmahi Ahmed:** writing – original draft, writing – review and editing. **Mehad Mortada BadrAlden Ahmed:** writing – original draft. **Aisha Gameraldeen Abdalrhim Ibrahim:** writing – original draft. **Arafa Mubarak Abotalib Aref:** writing – original draft. **Thowiba Mohammed Abdalla Saidahmed:** writing – original draft. **Osman Elshazali Osman Abd Elaziz:** writing – original draft. **Zahra Abdalla Ahmed Neel:** writing – original draft. **Mohammed Musa Abozaid Alkarar:** writing – original draft. **Tarig Mohamed Nourallah Altegani:** writing – review and editing. **Amjed Abdu Ali Mohammed:** writing – review and editing. **Mohammed Hammad Jaber Amin:** supervision.

## Ethics Statement

This study is exempt from ethical approval in our hospital.

## Consent

Written informed consent was obtained from the legal guardians of both twin sisters for publication of anonymized medical data, images, and genetic findings in accordance with the journal's policy. The guardians acknowledged the report's public availability and confirmed that all identifiable information has been removed to protect the patients' privacy.

## Conflicts of Interest

The authors declare no conflicts of interest.

## Data Availability

The data that support the findings of this study are available on request from the corresponding author. The data are not publicly available due to privacy or ethical restrictions.
